# Accelerometer-Measured Moderate-to-Vigorous Physical Activity and Cancer Risk: Dose–Response from Observational and Nonlinear Mendelian Randomization in UK Biobank

**DOI:** 10.3390/healthcare14131818

**Published:** 2026-06-23

**Authors:** Chang-Ling Huang, Meng-Xuan Yang, Yong-Qiao He, Wen-Qiong Xue, Ying Liao, Tong-Min Wang, Wei-Hua Jia

**Affiliations:** 1 School of Public Health, Sun Yat-sen University, Guangzhou 510080, China; huangchling3@mail2.sysu.edu.cn (C.-L.H.); yangmx25@mail2.sysu.edu.cn (M.-X.Y.); 2 State Key Laboratory of Oncology in South China, Collaborative Innovation Center for Cancer Medicine, Sun Yat-sen University Cancer Center, Guangzhou 510060, China; heyq@sysucc.org.cn (Y.-Q.H.); xuewq@sysucc.org.cn (W.-Q.X.); liaoying@sysucc.org.cn (Y.L.)

**Keywords:** physical activity, moderate-to-vigorous physical activity (MVPA), cancer incidence, Mendelian randomization, nonlinear dose–response

## Abstract

**Highlights:**

**What are the main findings?**

- Higher accelerometer-measured MVPA was associated with lower total cancer risk and multiple site-specific cancers.
- Nonlinear MR analyses suggested a potential threshold-like pattern, with an approximate inflection point around 5 h per week.

**What are the implications of the main findings?**

- By combining device-based measurement with nonlinear Mendelian randomization, this study provides causal-supportive evidence that refines the understanding of the MVPA-cancer dose–response relationship.
- The observed pattern may help guide future investigations of dose–response relationships between MVPA and cancer risk.

**Abstract:**

**Background:** Physical activity is a well-established modifiable lifestyle factor associated with reduced cancer risk; however, the optimal weekly volume of moderate-to-vigorous physical activity (MVPA) for cancer prevention, particularly when assessed using objective measures, remains unclear. Most existing evidence relies on self-reported physical activity, which may introduce measurement bias and obscure accurate dose–response relationships. **Methods:** We analyzed data from UK Biobank participants with valid accelerometer measurements to quantify habitual MVPA. Observational associations between MVPA and incident cancer were evaluated using multivariable Cox proportional hazards regression and restricted cubic splines. One-sample Mendelian randomization (MR) analyses, including both linear and nonlinear approaches, were conducted to evaluate potential causal associations and explore possible dose–response patterns. **Results:** Higher MVPA was associated with lower total cancer risk (HR 0.971, 95% CI 0.954–0.988, *p* = 0.001). Consistent associations were observed for several site-specific cancers, particularly lung, colorectal, breast, kidney, and bladder cancer. MR analyses supported a directionally consistent association between genetically predicted MVPA and lower total cancer risk (HR 0.977, 95% CI 0.962–0.992, *p* = 0.002). Nonlinear MR analyses suggested a potential nonlinear association, with lower cancer risk observed at a model-derived exploratory point of approximately 5 h of weekly MVPA. **Conclusions:** These findings provide supportive evidence that higher accelerometer-measured MVPA is associated with lower total cancer risk and contribute to a better understanding of the dose–response relationship between MVPA and cancer incidence.

## 1. Introduction

Cancer remains a leading cause of morbidity and mortality worldwide, posing a substantial public health burden [1]. Physical inactivity is widely recognized as an important modifiable risk factor for multiple non-communicable diseases [2,3], including major cancers such as colorectal, lung, and breast cancer [4,5]. Nevertheless, despite the general consensus on its importance, evidence regarding the precise magnitude and shape of the association between physical activity and cancer risk remains inconsistent across observational studies and intervention settings. Important challenges persist, including heterogeneity in associations across cancer types, residual confounding, and differences in population characteristics across studies. Beyond confirming the overall association, identifying whether there exists a specific threshold of MVPA volume above which cancer risk is reduced remains an important but underexplored question.

Most existing evidence is based on self-reported physical activity questionnaires, which may not accurately capture total daily movement and are subject to recall and reporting bias, thereby contributing to uncertainty in estimated dose–response relationships [6,7,8]. Wearable accelerometers offer a more standardized and objective assessment of habitual activity, particularly moderate-to-vigorous physical activity (MVPA), defined as activity ≥3 metabolic equivalents of task (METs) [9]. However, accelerometer-based assessment also carries methodological considerations, including differences in device-specific cut-points, limited comparability across studies, and potential misclassification of activity intensity, which may complicate interpretation of the dose–response relationship. These issues, together with the inherent constraints of observational designs, underscore that the association between objectively measured MVPA and cancer risk warrants further investigation using complementary analytical approaches [9,10,11].

Several prospective cohort studies have reported that higher physical activity levels were associated with reduced cancer incidence and mortality [10,11]. In a recent UK Biobank accelerometer-based analysis, Shreves et al. reported that higher levels of total physical activity, light-intensity physical activity, and MVPA were inversely associated with incident cancer, and observed a plateau in the step count-cancer association at approximately 9000 steps per day [9]. However, that analysis relied on categorizing activity into quintiles and did not employ nonlinear MR methods to explore the dose–response relationship from a causal inference perspective, leaving the question of whether a specific MVPA threshold exerts a causal effect on cancer risk unanswered.

To address this gap, Mendelian randomization (MR) employs genetic variants as instruments for long-term differences in physical activity and may help reduce bias from residual confounding and reverse causation [12,13]. While most MR studies have assumed linear exposure-outcome relationships [14], this assumption may be overly simplistic for behavioral exposures such as physical activity, where health effects may vary across activity levels [3,15]. For behavioral exposures such as physical activity, the relationship with health outcomes may not be strictly linear, and linear models may therefore obscure important features of the dose–response curve. Nonlinear MR approaches allow the investigation of potential nonlinear dose–response patterns and threshold-like features, thereby providing a more flexible framework for evaluating the relationship between physical activity and cancer risk.

In this study, we used UK Biobank data to examine the association between accelerometer-derived MVPA and incident cancer, with a specific focus on identifying potential threshold effects in the dose–response relationship, using multivariable Cox regression together with linear and nonlinear MR analyses to provide complementary evidence from both observational and causal inference perspectives.

## 2. Materials and Methods

### 2.1. Study Design, Data Source, and Participants

The UK Biobank is a population-based prospective cohort of 501,936 adults aged 40–69 years at recruitment [16]. Baseline data, including lifestyle and health-related information, were collected through questionnaires and physical measurements. A subset of participants underwent accelerometer-based physical activity assessment between 2013 and 2015 (*n* = 103,567). After excluding those missing or implausible accelerometer data (*n* = 8589), withdrawal of consent (*n* = 43), or cancer prior to assessment (*n* = 6379), 88,556 participants were included in the analytical sample. Only participants with valid accelerometer-derived MVPA and complete covariate data were included.

### 2.2. Physical Activity Assessment

Physical activity was assessed using wrist-worn Axivity AX3 accelerometers (Axivity Ltd., Newcastle upon Tyne, UK) worn continuously for 7 consecutive days [17]. MVPA was derived using the pre-computed UK Biobank accelerometer-based variable, generated according to the standard UK Biobank processing pipeline for wrist-worn accelerometer data. This variable represents the average weekly duration of MVPA accumulated over the 7-day monitoring period.

### 2.3. Outcome Ascertainment

Incident cancer cases were ascertained via the National Health Service Central Register and classified using ICD-9 and ICD-10 codes. Cancers diagnosed up to 20 February 2024 were included. Follow-up time was defined as the period from accelerometer measurement to the earliest of death, first cancer diagnosis, loss to follow-up, or study end. To ensure statistical stability, site-specific analyses were conducted for cancer types with ≥100 incident cases, resulting in 15 cancer types: head and neck, oesophagus, colorectum, pancreas, lung, melanoma, breast, endometrium, ovary, prostate, kidney, bladder, non-Hodgkin lymphoma, multiple myeloma, and leukaemia (Supplementary Table S1). Both Cox regression and MR analyses were performed for total and site-specific cancers.

### 2.4. Covariate Assessment

Covariates included demographic, lifestyle, and dietary factors. Age, sex, and deprivation index were obtained from UK Biobank records, while other variables were self-reported. For site-specific cancers, additional established risk factors were included, such as the four-level frequency of sun/UV protection use for melanoma, and hormone replacement therapy, oral contraceptive use, and reproductive factors for female-specific cancers. Only participants with complete covariate information were included, resulting in a complete-case analysis.

### 2.5. Observational Analysis

Baseline characteristics were summarized using means and standard deviations for continuous variables, and counts and percentages for categorical variables. Associations between weekly accelerometer-assessed MVPA and incident total cancer were evaluated using Cox regression, with hazard ratios (HRs) and 95% confidence intervals (CIs) reported. Covariates were selected based on prior evidence as potential confounders and introduced sequentially, with age and sex in minimally adjusted models, and additional adjustment for ethnicity, deprivation index, education, smoking, alcohol consumption, and dietary factors in fully adjusted models. Site-specific models further included established risk factors for specific cancers, such as sun/UV protection use for melanoma and hormone replacement therapy, oral contraceptive use, reproductive factors, and hysterectomy status for female-specific cancers. The proportional hazards assumption was evaluated using Schoenfeld residuals, and no violations were detected. To minimize reverse causation, cases diagnosed within the initial two years of follow-up were omitted in sensitivity analyses. Restricted cubic spline (RCS) models with four knots (5th, 35th, 65th, and 95th percentiles) were fitted using the survival and rms packages in R to assess the nonlinear relationship, with nonlinearity tested via likelihood ratio tests. To account for multiple comparisons across site-specific cancers, false discovery rate (FDR) correction was applied using the Benjamini–Hochberg procedure, and adjusted *p* values (*P_FDR_*) were reported.

### 2.6. Genetic Samples and Genotype Quality Control

Genetic analyses were conducted among participants with available genotype and accelerometer measurements. Comprehensive quality control (QC) procedures were implemented at participant and variant levels. Participants were excluded for insufficient genotype quality, outlier heterozygosity or missingness, non-European ancestry, aneuploid sex chromosomes, or discordant phenotypic and genotypic sex. Related individuals (kinship coefficient >0.0442) were excluded using KING software (version 2.3.1; RRID:SCR_009251), and the largest set of unrelated individuals was retained using the igraph package (version 2.1.4) in R. The final MR sample included 83,588 participants, representing a subset of the observational cohort. At the variant level, single-nucleotide polymorphisms (SNPs) were excluded for minor allele frequency (MAF) <0.01, Hardy–Weinberg equilibrium (*p* < 1 × 10^−12^), missingness > 0.05, or imputation INFO score < 0.8.

### 2.7. Genome-Wide Association Analyses (GWAS)

GWAS were conducted using UK Biobank individual-level data [16]. For total cancer, logistic regression was used to compare incident cases with cancer-free controls. MVPA was analyzed as a continuous trait using linear regression under an additive genetic model, adjusted for genotyping array, the first ten principal components, age, and sex [18].

### 2.8. Genetic Instruments and Mendelian Randomization

This study followed the Strengthening the Reporting of Observational Studies in Epidemiology Using Mendelian Randomization (STROBE-MR) statement [19,20] (Supplementary Table S2).

SNPs associated with MVPA (*p* < 1 × 10^−6^) were selected and clumped for linkage disequilibrium (r^2^ < 0.001 within a 1 Mb window). A weighted genetic risk score (GRS) based on GWAS effect estimates was used as the instrumental variable. Internal weights were applied due to the absence of large external GWAS with comparable phenotyping. All selected instruments had F-statistics >10 (Supplementary Tables S3 and S4), indicating adequate instrument strength. The proportion of variance explained (R^2^) was also calculated to quantify instrument strength and complement F-statistics. Of note, several selected variants have previously been associated with physical activity phenotypes in independent studies [21], including rs1243194 (Beta = 0.026, *p* = 5.8 × 10^−7^), rs12993139 (Beta = −0.014, *p* = 8.7 × 10^−3^), and rs970786 (Beta = 0.056, *p* = 1.1 × 10^−3^), providing external support for the relevance of the selected instruments.

A one-sample MR framework was adopted to ensure consistency between exposure, outcome, and covariate definitions within the same population and to enable nonlinear MR analyses, which require individual-level data. MR analyses were conducted for the total and the 15 site-specific cancers in the UK Biobank, applying both linear and nonlinear approaches. Linear MR estimates were performed using two-stage residual inclusion (2SRI) Cox regression [22] to estimate HRs for MVPA. To assess robustness of the estimates, Bayesian Markov chain Monte Carlo (MCMC) models [23] were additionally fitted using the same genetic risk score. SNP-level MR sensitivity analyses were performed by calculating Wald ratio estimates for each variant, which were combined using inverse-variance weighted (IVW) regression. Weighted median and MR-Egger regression were applied as complementary estimators. Horizontal pleiotropy was assessed using the MR-Egger intercept, and heterogeneity was quantified using Cochran’s Q statistic.

Nonlinear MR analyses were conducted using the nlmr package (version 1.0.3) in R, with participants stratified into 10 quantiles of the IV-free exposure derived from the residual method. Localized average causal effects (LACE) were estimated within each stratum, and fractional polynomial models were fitted to the stratum-specific estimates to characterize the dose–response relationship. 95% CIs were obtained from 1000 bootstrap iterations [24,25]. Given that the estimated HRs were above 1 at lower exposure levels and dropped below 1 at higher levels, an approximate crossing point was defined as the exposure level at which the fitted fractional polynomial curve first crossed the null (HR = 1). This value was treated as a model-based exploratory reference point rather than a definitive biological cutoff. The fractional polynomial specification was selected based on model fit criteria, and the stability of the estimated crossing point was evaluated using bootstrap resampling as described above.

### 2.9. Stratified Analyses

Stratified analyses were performed for Cox regression and MR models to examine potential effect modification across demographic and lifestyle subgroups. Participants were stratified by age group (<50, 50–60, ≥60 years), sex (male, female), BMI (non-obese, obese), and smoking status (never smoker, ever smoker), as these factors are associated with both physical activity and cancer risk.

Statistical analyses were performed using R version 4.4.3.

## 3. Results

Table 1 presents baseline characteristics for the 88,556 participants by tertiles of accelerometer-measured MVPA. Throughout an average follow-up of 8.6 years (median 9.1; interquartile range 8.5–9.7), 9088 incident cancer events were documented. The mean age was 55.6 years, and 55.6% were female. Participants in the lowest MVPA tertile had higher BMI and higher proportions of current smokers and alcohol drinkers.

### 3.1. Observational Associations of MVPA with Cancer Risk

**Figure 1:**
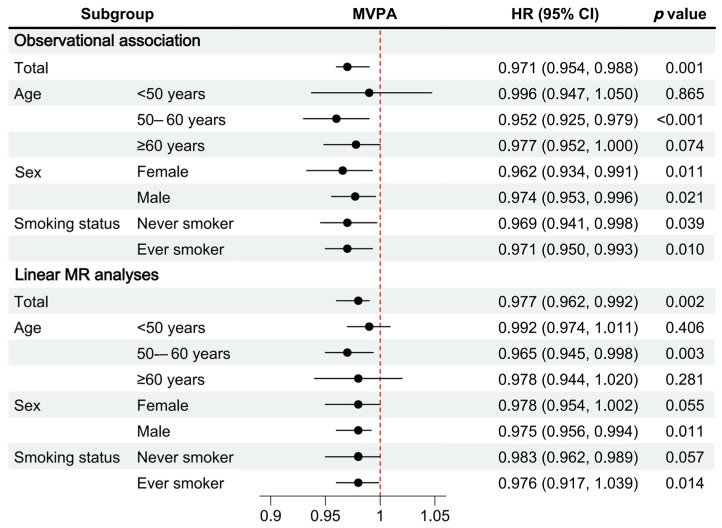
Associations of MVPA with total cancer incidence in observational and linear MR analyses. The dashed red line indicates HR = 1.

In the fully adjusted Cox model, accelerometer-measured MVPA was associated with a lower risk of total cancer (Figure 1, Supplementary Table S5). Each 1-SD (204.98 min) increment in MVPA was associated with a 2.6% lower risk of total cancer (HR 0.971, 95% CI 0.954–0.988, *p* = 0.001). Site-specific analyses indicated generally consistent associations (Supplementary Table S6), including colorectal (HR 0.858, 95% CI 0.779–0.946, *p* = 0.002, *P_FDR_* = 0.014), lung (HR 0.817, 95% CI 0.694–0.962, *p* = 0.015, *P_FDR_* = 0.049), breast (HR 0.857, 95% CI 0.794–0.925, *p* < 0.001, *P_FDR_* = 0.001), kidney (HR 0.768, 95% CI 0.651–0.905, *p* = 0.002, *P_FDR_* = 0.014), bladder cancer (HR 0.785, 95% CI 0.656–0.940, *p* = 0.009, *P_FDR_* = 0.034), and non-Hodgkin lymphoma (HR 0.696, 95% CI 0.545–0.888, *p* = 0.004, *P_FDR_* = 0.018). In contrast, a nominal positive association was observed for melanoma (HR 1.087, 95% CI 1.003–1.178, *p* = 0.041), which was not statistically significant after FDR correction (*P_FDR_* = 0.100). Similar non-significant findings were observed for multiple myeloma (HR 1.048, 95% CI 1.000–1.099, *p* = 0.052, *P_FDR_* = 0.119). Associations were broadly consistent across subgroups (Figure 1, Supplementary Table S5). A significant association was observed among participants aged 50–60 years (HR 0.952, 95% CI 0.925–0.979, *p* < 0.001), but not in those aged <50 years (*p* = 0.865) or ≥60 years (*p* = 0.074). Associations were significant in both females (HR 0.962, 95% CI 0.934–0.991, *p* = 0.011) and males (HR 0.974, 95% CI 0.953–0.996, *p* = 0.021). An association was observed in the non-obese participants (HR 0.973, 95% CI 0.954–0.993, *p* = 0.007), but not significant in obese participants (*p* = 0.303). Similar associations were observed in both ever smokers (HR 0.971, 95% CI 0.950–0.993, *p* = 0.010) and never smokers (HR 0.969, 95% CI 0.941–0.998, *p* = 0.039). Results were robust after excluding cancers diagnosed within the initial two years of follow-up (Supplementary Tables S7 and S8). The proportional hazards assumption was not violated for the Cox models.

### 3.2. Linear MR Analyses of MVPA and Cancer Risk

MR analyses supported the observational findings, with genetically predicted MVPA associated with a lower risk of total cancer. Each 1-SD increase in MVPA was associated with a 2.3% lower risk of total cancer (HR 0.977, 95% CI 0.962–0.992, *p* = 0.002) (Figure 1). Sensitivity analyses generally supported the robustness of the primary findings. There was no evidence of directional horizontal pleiotropy for total cancer (MR-Egger intercept *p* = 0.140), although significant heterogeneity was detected among the genetic instruments (Cochran’s Q = 132.8, *p* < 0.001) (Supplementary Table S9). Bayesian Mendelian randomization yielded consistent estimates, showing an inverse association between genetically predicted MVPA and total cancer risk (HR 0.997, 95% CI 0.994–0.999). At the SNP level, the IVW method confirmed the primary result (HR 0.997, 95% CI 0.996–0.998, *p* < 0.001), which was further supported by the weighted median estimator (HR 0.925, 95% CI 0.878–0.975, *p* = 0.004). In contrast, the MR-Egger method yielded a directionally consistent but statistically non-significant estimate (HR 1.011, 95% CI 0.934–1.095, *p* = 0.148) (Supplementary Table S10).

Statistically significant associations were observed in lung (HR 0.736, 95% CI 0.647–0.839, *p* < 0.001, *P_FDR_* = 0.001), kidney (HR 0.714, 95% CI 0.634–0.805, *p* < 0.001, *P_FDR_* = 0.001), and bladder cancer (HR 0.704, 95% CI 0.640–0.775, *p* < 0.001, *P_FDR_* < 0.001) (Supplementary Table S11). Directionally consistent associations were also observed for colorectal (HR 0.926, 95% CI 0.868–0.987, *p* = 0.018, *P_FDR_* = 0.054), breast (HR 0.960, 95% CI 0.926–0.995, *p* = 0.024, *P_FDR_* = 0.061), and oesophagus cancer (HR 0.528, 95% CI 0.312–0.895, *p* = 0.018, *P_FDR_* = 0.054). In contrast, melanoma (HR 1.056, 95% CI 0.985–1.131, *p* = 0.126, *P_FDR_* = 0.236) and non-Hodgkin lymphoma (HR 1.262, 95% CI 0.883–1.803, *p* = 0.201, *P_FDR_* = 0.302) showed directionally positive but statistically non-significant associations. Subgroup analyses showed generally consistent associations (Figure 1). Statistically significant associations were identified in participants aged 50–60 years (HR 0.965, 95% CI 0.945–0.998, *p* = 0.003), but not in those <50 or ≥60 years (*p* = 0.406 and *p* = 0.281, respectively). Similar patterns were observed by sex, BMI, and smoking status, with variation in statistical significance across strata. By sex, the effect was significant in males (HR 0.975, 95% CI 0.956–0.994, *p* = 0.011) and showed a similar trend in females (*p* = 0.055). Associations were observed in non-obese participants (HR 0.983, 95% CI 0.962–0.989, *p* = 0.003), but not in obese individuals (*p* = 0.453). Among ever smokers, MVPA was associated with lower cancer risk (HR 0.982, 95% CI 0.962–0.997, *p* = 0.014), and directionally similar in never smokers (*p* = 0.057).

### 3.3. Nonlinear MR of MVPA and Cancer Risk

Nonlinear MR analyses suggested a potential nonlinear association between MVPA and cancer risk, with a model-derived crossing point estimated around 289 min per week (95% CI: 288–290; Figure 2). Formal tests for non-linearity were not statistically significant for most cancer types (Supplementary Table S11), indicating that the evidence for a nonlinear shape was limited. Subgroup analyses showed broadly similar patterns across strata defined by age, smoking status, sex, and BMI, with point estimates generally consistent with the main analysis and no clear evidence of effect modification (Supplementary Figure S3).

## 4. Discussion

Our analysis suggested that higher MVPA levels were associated with lower overall cancer risk, with broadly consistent associations observed across key demographic and lifestyle subgroups. One-sample MR results were directionally consistent with the observational findings, while nonlinear MR suggested an approximate model-derived crossing point around 5 h per week, which should be interpreted cautiously. These findings are in line with previous observational studies and meta-analyses suggesting a reduced risk between physical activity and total cancer risk, with prior cohort and pooled analyses reporting risk reductions of approximately 10–12% [26]. Accelerometer-based assessments may provide a more comprehensive assessment of activity, capturing both structured and incidental movement that is often underreported in questionnaires. However, the 7-day monitoring period may not fully reflect long-term habitual physical activity patterns across the life course. Although the observed effect size for total cancer incidence was modest, such small relative risk reductions may still have important public health implications at the population level.

Sensitivity analyses using multiple complementary MR approaches provided overall consistent evidence supporting the robustness of the primary findings. In particular, IVW and weighted median estimates were directionally consistent and statistically significant, and Bayesian MR produced similar effect estimates. Although significant heterogeneity was detected among genetic instruments for total cancer, no evidence of directional horizontal pleiotropy was observed based on the MR-Egger intercept, suggesting that the causal estimates were unlikely to be substantially biased by systematic pleiotropic effects.

Nonlinear MR analysis suggested a potential nonlinear association between MVPA and cancer risk, with a model-derived inflection-like pattern around 5 h per week. Evidence from prospective cohort studies also suggests that health benefits may vary across activity levels, which is broadly consistent with a potential nonlinear relationship [27]. However, this finding should be considered exploratory, and this pattern should be interpreted as a model-based feature rather than a definitive behavioral threshold [28]. Importantly, MVPA was derived as a total weekly volume from the UK Biobank summary pipeline and could not be further separated into bouted and non-bouted activity. Therefore, this threshold should be interpreted with caution, as continuous activity bouts and sporadic short-duration activities may impose distinct physiological stimuli. Sustained efforts primarily affect cardiorespiratory adaptation, whereas fragmented activities predominantly influence metabolic regulation. Future studies incorporating bout-specific accelerometer measures are warranted to further clarify this relationship. From a public health perspective, approximately half of the participants were below this level, indicating substantial variability in physical activity across the cohort.

Higher MVPA was generally associated with lower cancer risk for several site-specific cancers. Consistent with previous evidence [14,29], lower risks were observed for breast and colorectal cancers. We also observed potential associations for bladder and oesophageal cancers, consistent with prior systematic reviews and meta-analyses [30]. A significant association was also identified for lung cancer, despite limited prior evidence. Overall, most site-specific associations were directionally consistent between observational and MR analyses. However, discrepancies were observed for some cancer types. For example, non-Hodgkin lymphoma suggested a lower risk in observational analyses but a directionally positive, non-significant association in MR analyses. This discrepancy may reflect differences in study design, with observational analyses capturing short-term measured behavior, whereas MR reflects lifelong genetic predisposition. Site-specific MR analyses were limited by small numbers of cases for certain cancers, reducing statistical power and contributing to wide confidence intervals; therefore, individual cancer findings should be interpreted cautiously. In contrast, higher MVPA was associated with an increased risk of melanoma, consistent with a previous meta-analysis [31], although this association did not survive multiple testing correction, and should therefore be considered exploratory. We adjusted for the frequency of sun/UV protection use as a proxy for UV-related behavior; however, this does not capture cumulative UV exposure or time outdoors, which were unavailable in UK Biobank. Importantly, if higher MVPA reflects greater outdoor time, unmeasured UV exposure would act as a positive confounder, biasing the hazard ratio away from the null and overestimating the true MVPA effect. Thus, this association should be interpreted cautiously, as it may be partially driven by unmeasured sun exposure rather than a direct causal effect.

Subgroup analyses showed broadly consistent associations between MVPA and total cancer risk across most demographic and lifestyle strata, supporting the robustness of the main observational and MR findings. While point estimates varied somewhat across subgroups defined by age, sex, BMI, and smoking status, formal interaction tests were not performed to statistically compare these estimates across strata. Consequently, these subgroup-specific findings should be interpreted as strictly exploratory and do not support conclusions regarding differential associations or effect modification by these factors. The consistently positive overall association across most groups remains the primary focus of our interpretation.

Several limitations should also be considered. First, the study population was restricted to individuals of European ancestry, which reduces population stratification bias but may limit generalizability to other populations. Differences in genetic architecture, environmental exposures, and physical activity patterns, together with the healthier and more health-conscious nature of the UK Biobank cohort compared with the general population, further limit external validity. Therefore, caution is warranted when extrapolating these findings to global populations, and replication in more diverse ancestry groups is needed. In addition, the complete-case design may also introduce selection bias, as participants with complete accelerometer data were systematically healthier than the full cohort. Second, this study used a one-sample MR design, as external GWAS summary statistics specific to accelerometer-derived MVPA are not available. Existing self-reported physical activity GWAS differ substantially in phenotype definition and would have introduced heterogeneity. The one-sample design may introduce bias due to sample overlap, including winner’s curse, overfitting, and weak instrument bias. Although all instruments had F-statistics >10 and sensitivity analyses were performed, residual bias and effect-size inflation cannot be excluded. Third, to enhance statistical power, we employed a relaxed significance threshold (*p* < 1 × 10^−6^) for instrument selection. While this strategy increases the number of available instruments, it may also have allowed the inclusion of weaker variants, thereby introducing weak-instrument bias. Although sensitivity analyses using alternative MR approaches yielded consistent results, residual uncertainty cannot be fully excluded. Finally, several site-specific cancer analyses had limited case numbers, reducing statistical power. Residual confounding may remain from occupational physical activity (which may differ from leisure-time MVPA), as well as unmeasured sedentary behavior, sleep, comorbidities, medication use, and overall diet.

## 5. Conclusions

Our study provides convergent evidence from observational and MR analyses that higher accelerometer-derived MVPA was associated with lower cancer incidence. A possible model-derived crossing point was observed at approximately 5 h per week, although the effect sizes were relatively modest. Associations were generally consistent across subgroups. From a public health perspective, these findings underscore the population-level benefits of promoting MVPA.

## Figures and Tables

**Figure 2:**
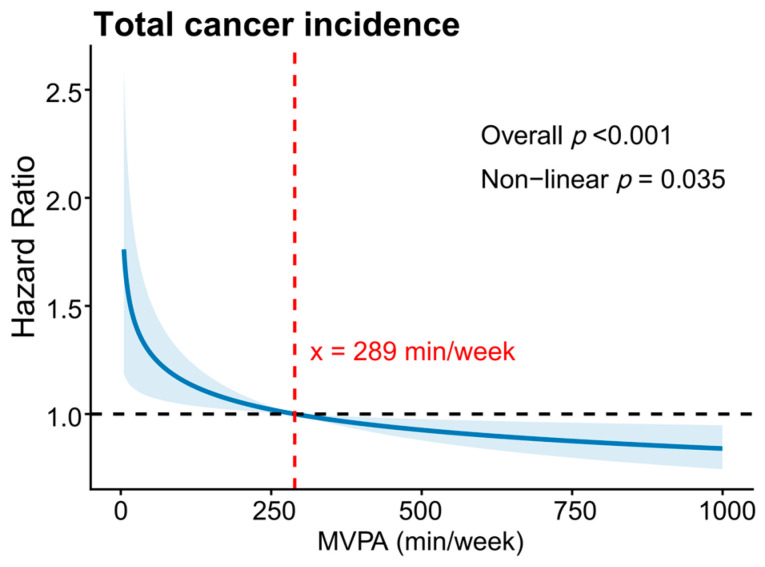
Nonlinear MR of the association between MVPA and total cancer incidence. The solid line represents the HR, and the shaded area indicates the 95% CI. The horizontal dashed line denotes the reference level (HR = 1.0). The vertical dashed line indicates the approximate model-derived crossing point at 289 min per week derived from the model-based estimation.

**Table 1:** Baseline characteristics of participants according to tertiles of MVPA (min/week).

	MVPA (min/Week)			
	Total	Low (<150 min/Week)	Medium (150–299 min/Week)	High (≥300 min/Week)
Total participants	88,556	31,226	24,157	33,173
Age at baseline (mean/SD)	55.55 (7.85)	56.26 (7.88)	55.59 (7.86)	54.86 (7.75)
Sex				
Female	49,206 (55.6)	20,216 (64.7)	14,087 (58.3)	14,903 (44.9)
Male	39,350 (44.4)	11,010 (35.3)	10,070 (41.7)	18,270 (55.1)
Ethnicity				
Other	3364 (3.8)	1369 (4.4)	928 (3.8)	1067 (3.2)
White	85,192 (96.2)	29,857 (95.6)	23,229 (96.2)	32,106 (96.8)
Deprivation Index (mean/SD)	14.95 (12.06)	15.79 (12.73)	14.78 (11.89)	14.30 (11.47)
Education *				
University degree	38,453 (43.4)	11,100 (35.5)	10,532 (43.6)	16,821 (50.7)
A-levels/HNC/HND/NVQ	21,633 (24.4)	8006 (25.6)	5935 (24.6)	7692 (23.2)
GCSE/O-level/CSE	16,288 (18.4)	6785 (21.7)	4494 (18.6)	5009 (15.1)
Other	4722 (5.3)	1932 (6.2)	1255 (5.2)	1535 (4.6)
None	7460 (8.4)	3403 (10.9)	1941 (8.0)	2116 (6.4)
BMI, kg/m^2^ (mean/SD)	26.74 (4.55)	28.01 (5.20)	26.52 (4.24)	25.71 (3.77)
BMI, kg/m^2^				
Non-obese (BMI < 30 kg/m^2^)	70,463 (80.3)	21,989 (70.9)	19,578 (81.7)	28,896 (87.9)
Obese (BMI ≥ 30 kg/m^2^)	17,389 (19.7)	9005 (29.1)	4396 (18.3)	3988 (12.1)
Smoking status				
Never smoker	35,559 (40.3)	12,237 (39.3)	10,012 (41.6)	13,310 (40.2)
Ever smoker	52,767 (59.7)	18,902 (60.7)	14,082 (58.4)	19,783 (59.8)
Alcohol intake				
Never drinker	2596 (2.9)	1158 (3.9)	786 (2.7)	652 (2.2)
Ever drinker	85,875 (97.1)	29,976 (96.1)	23,493 (97.3)	32,406 (97.8)

* Education categories were defined according to UK Biobank classifications. A-levels: Advanced level qualifications; HNC: Higher National Certificate; HND: Higher National Diploma; NVQ: National Vocational Qualification; GCSE: General Certificate of Secondary Education; O-level: Ordinary level qualification; CSE: Certificate of Secondary Education.

## Data Availability

The data analyzed in this study are available from the UK Biobank Resource under Application Number 58450. Access to the data is available to bona fide researchers upon application to the UK Biobank (http://www.ukbiobank.ac.uk/) and subject to approval by the UK Biobank Access Management Team. Restrictions apply to the availability of these data, which were used under license for the current study and are therefore not publicly available.

## Supplementary Materials

The following supporting information can be downloaded at: https://www.mdpi.com/article/10.3390/healthcare14131818/s1. Figure S1: Association between MVPA and incident total cancer based on restricted cubic spline Cox regression. The solid line represents the adjusted HR. The model was adjusted for age, sex, ethnicity, education, deprivation index, fruit and vegetable intake, smoking status and alcohol intake; Figure S2: Association between MVPA and incident total cancer based on restricted cubic spline Cox regression across subgroups. Line plots illustrate the adjusted hazard ratios for incident total cancer according to MVPA volume stratified by (A) age groups, (B) sex, (C) BMI categories, and (D) smoking status. All models were adjusted for the same covariates as in Supplementary Figure S1, except for the stratification variable; Figure S3: Associations of MVPA volume with the risk of total cancer across subgroups. Line plots illustrate the adjusted hazard ratios for incident total cancer according to MVPA volume stratified by (A) age groups, (B) sex, (C) BMI categories, and (D) smoking status; Table S1: Definitions of cancer outcomes and corresponding ICD-9 and ICD-10 codes; Table S2: STROBE-MR checklist of recommended items to address in reports of Mendelian randomization studies; Table S3: Summary information of each SNP used as instrument variants for both linear and non-linear Mendelian randomization analysis; Table S4: Associations of MVPA instrumental SNPs with cancer outcomes in UK Biobank pan-cancer GWAS; Table S5: Associations between physical activity (per SD increase) and risk of incident total cancer in Cox proportional hazards models; Table S6: Associations between physical activity (per SD increase) and site-specific cancer risk in Cox models; Table S7: Associations between physical activity (per SD increase) and incident total cancer in primary and 2-year lag Cox proportional hazards models; Table S8: Associations between physical activity (per SD increase) and site-specific cancer risk in primary and 2-year lag Cox proportional hazards models; Table S9: Assessment of horizontal pleiotropy and heterogeneity in Mendelian randomization analyses; Table S10: Sensitivity analyses for the causal effect of MVPA on total cancer risk using alternative Mendelian randomization methods; Table S11: Linear mendelian randomization estimates for analysis of MVPA on specific cancer incidence; Table S12. Nonlinear Mendelian randomization estimates for MVPA and site-specific cancer incidence.

## Abbreviations

The following abbreviations are used in this manuscript:

BMI	Body mass index
CI	Confidence interval
FDR	False discovery rate
GRS	Genetic risk score
GWAS	Genome-wide association study
HNC	Higher National Certificate
HND	Higher National Diploma
HR	Hazard ratio
ICD	International Classification of Diseases
IV	Instrumental variable
IVW	Inverse-variance weighted
LACE	Localized average causal effect
MET	Metabolic equivalent of task
MR	Mendelian randomization
MVPA	Moderate-to-vigorous physical activity
NHS	National Health Service
NVQ	National Vocational Qualification
O-level	Ordinary level qualification
QC	Quality control
RCS	Restricted cubic spline
SD	Standard deviation
SNP	Single-nucleotide polymorphism
STROBE-MR	Strengthening the Reporting of Observational Studies in Epidemiology using Mendelian Randomization
UKB	UK Biobank
UV	Ultraviolet
2SRI	Two-stage residual inclusion
