# Supplementary material for: Accelerometer-Measured Moderate-to-Vigorous Physical Activity and Cancer Risk: Dose–Response from Observational and Nonlinear Mendelian Randomization in UK Biobank
Source: Healthcare (Basel). 2026 Jun 23;14(13):1818. doi: 10.3390/healthcare14131818 (PMC13362087; doi:10.3390/healthcare14131818)
Supplement: Supplementary file 1 [file healthcare-14-01818-s001.zip › healthcare-4347943-supplementary.pdf]

Supplementary Material

Supplementary Figures and Tables

Supplementary Figures

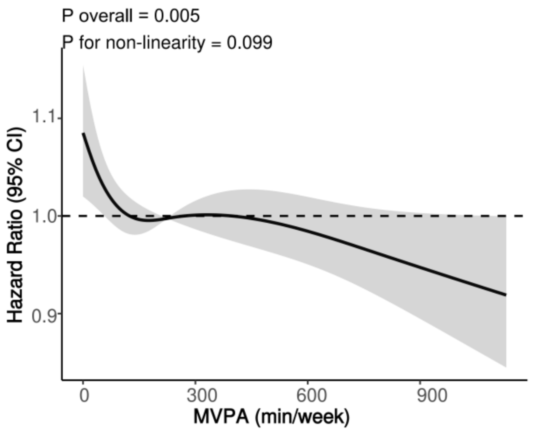

Supplementary Figure S1. Association between MVPA and incident total cancer based on restricted cubic spline Cox regression. The solid line represents the adjusted HR. The model was adjusted for age, sex, ethnicity, education, deprivation index, fruit and vegetable intake, smoking status and alcohol intake.

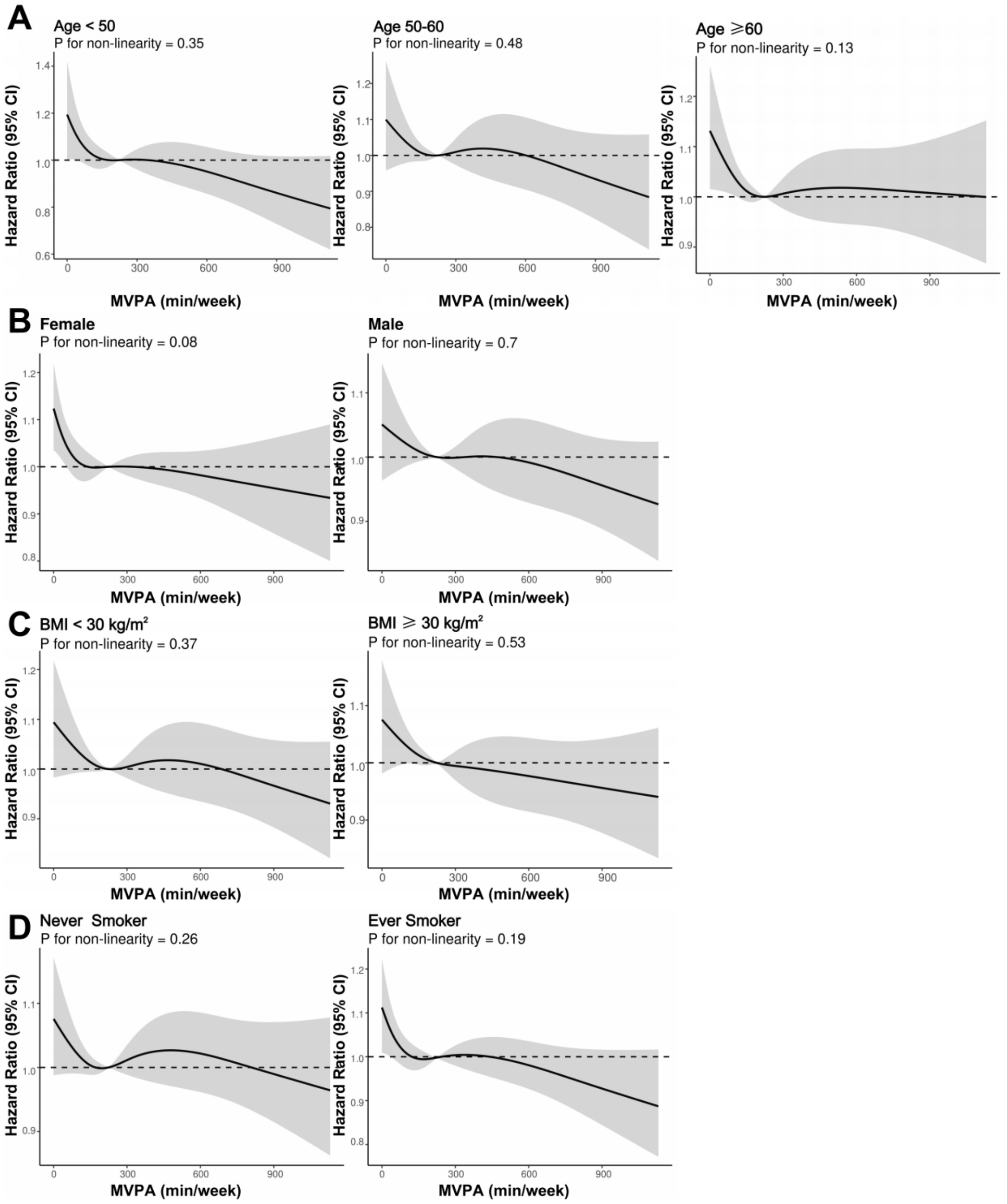

Supplementary Figure S2. Association between MVPA and incident total cancer based on restricted cubic spline Cox regression across subgroups. Line plots illustrate the adjusted hazard ratios for incident total cancer according to MVPA volume stratified by (A) age groups, (B) sex, (C) BMI categories, and (D) smoking status. All models were adjusted for the same covariates as in Supplementary Figure 1, except for the stratification variable.

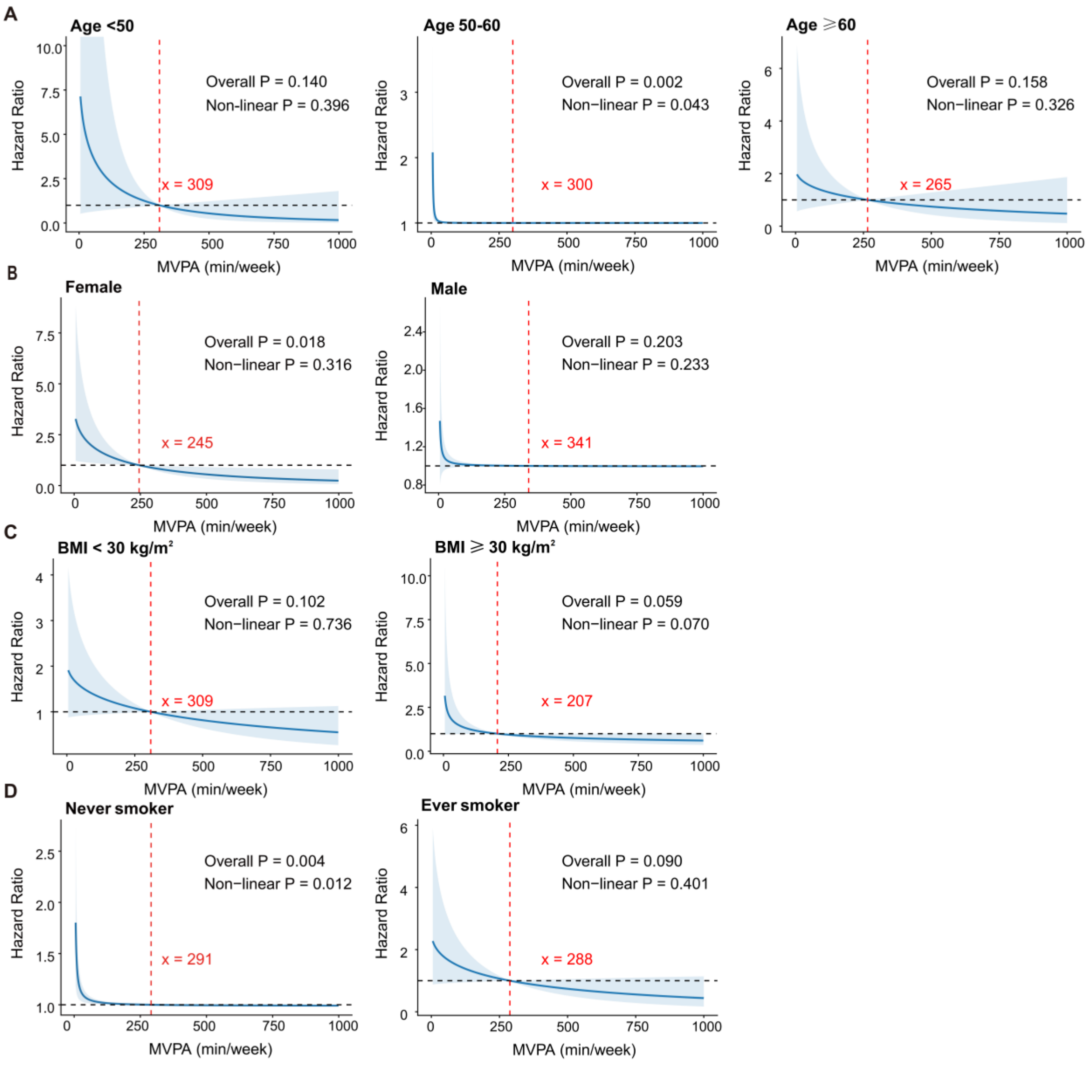

Supplementary Figure S3. Associations of MVPA volume with the risk of total cancer across subgroups. Line plots illustrate the adjusted hazard ratios for incident total cancer according to MVPA volume stratified by (A) age groups, (B) sex, (C) BMI categories, and (D) smoking status.

Supplementary Tables

Supplementary Table S1. Definitions of cancer outcomes and corresponding ICD-9 and ICD-10 codes.

Cancer Type	ICD-10	ICD-9
Head and neck	C00-C14, C30-C32	140-149, 161
Oesophagus	C15	150
Colorectum	C18-C20	153-154
Pancreas	C25	157
Lung	C33-C34	162
Melanoma	C43	172
Breast	C50	174
Endometrium	C54	182
Ovary	C56	183
Prostate	C61	185
Kidney	C64	189
Bladder	C67	188
Non-Hodgkin lymphoma	C82-C86	200-202
Multiple myeloma	C90	203
Leukaemia	C91-C95	204-208

Supplementary Table S2. STROBE-MR checklist of recommended items to address in reports of Mendelian randomization studies [1,2].

Item No.	Section	Checklist item	Page No.	Relevant text from manuscript
1	TITLE and ABSTRACT	Indicate Mendelian randomization (MR) as the study's design in the title and/or the abstract if that is a main purpose of the study	Page 1	"Mendelian randomization" in abstract
	INTRODUCTION			
2	Background	Explain the scientific background and rationale for the reported study. What is the exposure? Is a potential causal relationship between exposure and outcome plausible? Justify why MR is a helpful method to address the study question	Introduction	Concept of Mendelian randomization and specific request for Mendelian randomization were explained in the 3rd paragraph of the introduction.
3	Objectives	State specific objectives clearly, including pre-	Introduction	The causal question has been stated in the 3rd paragraph of the introduction.

		specified causal hypotheses (if any). State that MR is a method that, under specific assumptions, intends to estimate causal effects		
	METHODS			
4	Study design and data sources	Present key elements of the study design early in the article. Consider including a table listing sources of data for all phases of the study. For each data source contributing to the analysis, describe the following:		
	a)	Setting: Describe the study design and the underlying population, if possible. Describe the setting, locations, and relevant dates, including periods of recruitment,	Methods	Described in Methods "Study Design, Data Source, and Participants" section

		exposure, follow-up, and data collection, when available.		
	b)	Participants: Give the eligibility criteria, and the sources and methods of selection of participants. Report the sample size, and whether any power or sample size calculations were carried out prior to the main analysis	Methods	Described in Methods “Genetic Samples and Genotype Quality Control” section
	c)	Describe measurement, quality control and selection of genetic variants		
	d)	For each exposure, outcome, and other relevant variables, describe methods of assessment and diagnostic criteria for diseases	Methods	Incident cancer cases were ascertained via national cancer registries, and were coded according to ICD-9 and ICD-10. Other process has been described in the Methods section.
	e)	Provide details of ethics committee approval and	Ethics Approval and Consent to Participate	Ethical approval was provided by the UK National Health Service and National Research Ethics Service (Ethics

		participant informed consent, if relevant		Committee reference number: 11/NW/0382).
5	Assumptions	Explicitly state the three core IV assumptions for the main analysis (relevance, independence and exclusion restriction) as well assumptions for any additional or sensitivity analysis	Methods	Described in Methods "Genetic instruments and Mendelian randomization" section
6	Statistical methods: main analysis	Describe statistical methods and statistics used		
	a)	Describe how quantitative variables were handled in the analyses (i.e., scale, units, model)	Methods	Described in Methods "Assessment of covariates" and "Genetic instruments and Mendelian randomization" section
	b)	Describe how genetic variants were handled in the analyses and, if applicable, how their weights were selected		

	c)	Describe the MR estimator (e.g. two-stage least squares, Wald ratio) and related statistics. Detail the included covariates and, in case of two-sample MR, whether the same covariate set was used for adjustment in the two samples		
	d)	Explain how missing data were addressed		
	e)	If applicable, indicate how multiple testing was addressed		
7	Assessment of assumptions	Describe any methods or prior knowledge used to assess the assumptions or justify their validity	Methods	Described in Methods "Genetic instruments and Mendelian randomization" section.
8	Sensitivity analyses and additional analyses	Describe any sensitivity analyses or additional analyses performed (e.g. comparison of effect estimates from different approaches, independent	Methods	Described in Methods "Genetic instruments and Mendelian randomization" section.

		replication, bias analytic techniques, validation of instruments, simulations)		
9	Software and pre-registration			
	a)	Name statistical software and package(s), including version and settings used	Methods	All the statistical analysis tools used in this study are shown in "Genetic instruments and Mendelian randomization" section in Methods.
	b)	State whether the study protocol and details were pre-registered (as well as when and where)		
	RESULTS			
10	Descriptive data			
	a)	Report the numbers of individuals at each stage of included studies and reasons for exclusion. Consider use of a flow diagram	Results	All necessary information about the studies used in this study is described in the Results section; summary statistics are explained in Table 1.
	b)	Report summary statistics for phenotypic exposure(s),		

		outcome(s), and other relevant variables (e.g. means, SDs, proportions)		
	c)	If the data sources include meta-analyses of previous studies, provide the assessments of heterogeneity across these studies	Not applicable.	Not applicable.
	d)	For two-sample MR:<br>i. Provide justification of the similarity of the genetic variant-exposure associations between the exposure and outcome samples<br>ii. Provide information on the number of individuals who overlap between the exposure and outcome studies	Not applicable.	Not applicable.
11	Main results			
	a)	Report the associations between genetic variant and	Results	

		exposure, and between genetic variant and outcome, preferably on an interpretable scale	Supplementary Material	
	b)	Report MR estimates of the relationship between exposure and outcome, and the measures of uncertainty from the MR analysis, on an interpretable scale, such as odds ratio or relative risk per SD difference		Genetic exposure associations are reported in Figures 1-2, Supplementary Figure 2, and Supplementary Tables 9-11.<br>Our results were presented in terms of HR and confidence intervals throughout the Results section.
	c)	If relevant, consider translating estimates of relative risk into absolute risk for a meaningful time period		
	d)	Consider plots to visualize results (e.g. forest plot, scatterplot of associations between genetic variants and		

		outcome versus between genetic variants and exposure)		
12	Assessment of assumptions			
	a)	Report the assessment of the validity of the assumptions	Methods	Through the "Genetic instruments and Mendelian randomization" in the Methods section we ensured the reliability of the core assumptions.
	b)	Report any additional statistics (e.g., assessments of heterogeneity across genetic variants, such as I2, Q statistic or E-value)		
13	Sensitivity analyses and additional analyses			
	a)	Report any sensitivity analyses to assess the robustness of the main results to violations of the assumptions	Results	Described in Results “Linear MR of MVPA and cancer risk” and “Nonlinear MR of MVPA and cancer risk” section, Figure 1, Supplementary Figure 3, and Supplementary Tables 9-12. .
	b)	Report results from other sensitivity analyses or additional analyses		

	c)	Report any assessment of direction of causal relationship (e.g., bidirectional MR)		
	d)	When relevant, report and compare with estimates from non-MR analyses		
	e)	Consider additional plots to visualize results (e.g., leave-one-out analyses)		
	DISCUSSION			
14	Key results	Summarize key results with reference to study objectives	Discussion<br>Conclusion	Discussion paragraph 1.<br>Conclusion
15	Limitations	Discuss limitations of the study, taking into account the validity of the IV assumptions, other sources of potential bias, and imprecision. Discuss both direction and magnitude of any potential bias and any efforts to address them	Discussion	Discussion paragraph 6.

16	Interpretation			
	a)	Meaning: Give a cautious overall interpretation of results in the context of their limitations and in comparison with other studies	Discussion<br>Conclusion	Discussion paragraphs 2-6.<br>Conclusion
	b)	Mechanism: Discuss underlying biological mechanisms that could drive a potential causal relationship between the investigated exposure and the outcome, and whether the gene-environment equivalence assumption is reasonable. Use causal language carefully, clarifying that IV estimates may provide causal effects only under certain assumptions		
	c)	Clinical relevance: Discuss whether the results have		

		clinical or public policy relevance, and to what extent they inform effect sizes of possible interventions		
17	Generalizability	Discuss the generalizability of the study results (a) to other populations, (b) across other exposure periods/timings, and (c) across other levels of exposure	Discussion	We have discussed the potential caveats in terms of generalizability of our findings in Discussion.
	OTHER INFORMATION			
18	Funding	Describe sources of funding and the role of funders in the present study and, if applicable, sources of funding for the databases and original study or studies on which the present study is based	Acknowledgments	We have reported all sources of funding in the "Acknowledgments" section.
19	Data and data sharing	Provide the data used to perform all analyses or report where and how the data can be	Data Availability	The data used in this study are from UK Biobank, which can be accessed by researchers through an approved

		accessed, and reference these sources in the article. Provide the statistical code needed to reproduce the results in the article, or report whether the code is publicly accessible and if so, where		application process (https://www.ukbiobank.ac.uk), and the statistical code is available upon request from the corresponding author.
20	Conflicts of Interest	All authors should declare all potential conflicts of interest	Conflicts of Interest	The authors declare no conflicts of interest.

This checklist is copyrighted by the Equator Network under the Creative Commons Attribution 3.0 Unported (CC BY 3.0) license.

1. Skrivankova VW, Richmond RC, Woolf BAR, Yarmolinsky J, Davies NM, Swanson SA, et al. Strengthening the Reporting of Observational Studies in Epidemiology Using Mendelian Randomization: The STROBE-MR Statement. *JAMA* 2021;326(16):1614-21.
2. Skrivankova VW, Richmond RC, Woolf BAR, Davies NM, Swanson SA, VanderWeele TJ, et al. Strengthening the reporting of observational studies in epidemiology using mendelian randomisation (STROBE-MR): explanation and elaboration. *BMJ* 2021;375:n2233.

Supplementary Table S3. Summary information of each SNP used as instrument variants for both linear and non-linear Mendelian randomization analysis

SNP	CHR	POS	Effect Allele	Other Allele	EAF	beta	se	*P*	R^2	F
rs12993139	2	175186831	G	C	0.254	0.503	0.100	5.04E-07	0.0959	25.26
rs80038513	4	139326616	A	G	0.011	0.419	0.074	5.72E-08	0.0038	29.46
rs147357300	5	160667314	G	A	0.013	-0.113	0.024	4.35E-07	0.0004	25.53
rs71566494	6	148015373	G	A	0.023	0.380	0.081	1.80E-08	0.0065	31.69
rs1243194	10	21980514	A	G	0.288	0.431	0.092	3.88E-07	0.0762	25.76
rs970786	14	77480692	G	A	0.013	0.126	0.028	8.87E-07	0.0004	24.17

Supplementary Table S4. Associations of MVPA instrumental SNPs with cancer outcomes in UK Biobank pan-cancer GWAS

SNP	CHR	POS	Nearest Gene	Effect Allele	Other Allele	EAF	Beta	Se	***P***
rs12993139	2	175186831	SP9	G	C	0.254	0.0237	8e-05	7.1e-03
rs80038513	4	139326616	SLC7A11	A	G	0.011	-0.0987	1.2e-03	4.4e-03
rs147357300	5	160667314	GABRB2	G	A	0.013	0.0259	1.1e-03	4.4e-01
rs71566494	6	148015373	SAMD5	G	A	0.023	0.0206	6.1e-04	4.1e-01
rs1243194	10	21980514	MLLT10	A	G	0.288	-0.0279	7e-05	9.8e-04
rs970786	14	77480692	IRF2BPL	G	A	0.013	0.02391	1.1e-03	4.7e-1

Supplementary Table S5. Associations between physical activity (per SD increase) and risk of incident total cancer in Cox proportional hazards models

	Model 1: HR (95% CI)*	*P**	Model 2: HR (95% CI)†	*P*†
**Total**	0.974 (0.957, 0.990)	0.002	0.971 (0.954, 0.988)	0.001
**Age**				
<50 years	0.993 (0.945, 1.042)	0.763	0.996 (0.947, 1.050)	0.865
50-60 years	0.953 (0.927, 0.979)	0.001	0.952 (0.925, 0.979)	<0.001
≥60 years	0.983 (0.959, 1.007)	0.163	0.977 (0.952, 1.000)	0.074
**Sex**				
Female	0.964 (0.937, 0.992)	0.012	0.962 (0.934, 0.991)	0.011
Male	0.979 (0.958, 1.000)	0.051	0.974 (0.953, 0.996)	0.021
**BMI (Kg/m^2)**				
Non-obese	0.974 (0.956, 0.992)	0.005	0.973 (0.954, 0.993)	0.007
Obese	0.966 (0.921, 1.013)	0.152	0.974 (0.927, 1.024)	0.303
**Smoking status**				
Never	0.983 (0.956, 1.011)	0.239	0.969 (0.941, 0.998)	0.039
Ever smoker	0.969 (0.949, 0.990)	0.004	0.971 (0.950, 0.993)	0.010

* Model 1: adjusted for age and sex.

† Model 2: adjusted for age, sex, ethnicity (white/other), education (None, GCSE/O-level/CSE,A-levels/HNC/HND/NVQ, University degree, Other), deprivation index, fruit and vegetable intake, smoking status (never, former smoker, current smoker) and alcohol intake status (never, former drinker, current drinker).

Supplementary Table S6. Associations between physical activity (per SD increase) and site-specific cancer risk in Cox models

Cancer Type	Cases	HR per sd (95% CI)*	*P**	P_{FDR}*	HR per sd (95% CI)†	*P*†	P_{FDR}†
Head and neck	115	0.936 (0.776, 1.129)	0.490	0.562	0.970 (0.868, 1.084)	0.591	0.667
Oesophagus	115	0.697 (0.519, 0.937)	0.017	0.041	0.653 (0.434, 0.982)	**0.041**	0.100
Colorectum	613	0.856 (0.777, 0.943)	0.002	0.011	0.858 (0.779, 0.946)	**0.002**	**0.014**
Pancreas	154	0.899 (0.698, 1.158)	0.410	0.516	0.931 (0.787, 1.103)	0.409	0.515
Lung	317	0.761 (0.618, 0.936)	0.010	0.030	0.817 (0.694, 0.962)	**0.015**	**0.049**
Melanoma	145	1.106 (1.025, 1.194)	0.009	0.028	1.087 (1.003, 1.178) [a]	0.041	0.100
Breast	1,020	0.861 (0.799, 0.928)	<0.001	0.002	0.857 (0.794, 0.925) [b]	**<0.001**	**0.001**
Endometrium	158	0.821 (0.662, 1.018)	0.072	0.122	0.856 (0.692, 1.059) [b]	0.152	0.258
Ovary	106	0.679 (0.500, 0.922)	0.013	0.037	0.470 (0.231, 0.954) [b]	**0.037**	0.100
Prostate	1,384	1.055 (1.007, 1.105)	0.025	0.051	0.696 (0.472, 1.026)	0.067	0.138
Kidney	131	0.667 (0.527, 0.845)	0.001	0.008	0.768 (0.651, 0.905)	**0.002**	**0.014**
Bladder	116	0.784 (0.665, 0.925)	0.004	0.015	0.785 (0.656, 0.940)	**0.009**	**0.034**
Non-Hodgkin lymphoma	234	0.774 (0.658, 0.911)	0.002	0.011	0.696 (0.545, 0.888)	**0.004**	**0.018**
Multiple myeloma	101	0.719 (0.544, 0.950)	0.020	0.046	1.048 (1.000, 1.099)	0.052	0.119
Leukaemia	136	0.829 (0.670, 1.027)	0.086	0.139	0.819 (0.606, 1.107)	0.193	0.314

* Models adjusted for age and sex.

† Models adjusted for age, sex, ethnicity (white/other), education (None, GCSE/O-level/CSE,A-levels/HNC/HND/NVQ, University degree, Other), deprivation index, fruit and vegetable intake, smoking status (never, former smoker, current smoker) and alcohol intake status (never, former drinker, current drinker).

[a] Additional covariates specific to melanoma included frequency of sun/UV protection (never/rarely/sometimes, most of the time/always, do not go out in sunshine).

[b] Additional covariates specific to breast cancer included HRT use (ever/never used), oral contraceptive use (ever/never used), number of live births, age at menarche (<12, 12–14, ≥15 years), age at menopause (<45, 45–55, ≥55 years, not yet/unsure), hysterectomy status.

Supplementary Table S7. Associations between physical activity (per SD increase) and incident total cancer in primary and 2-year lag Cox proportional hazards models

	**Model 1: HR (95% CI)***	***P****	**Model 2: HR (95% CI)†**	***P*†**
**Total**	0.974 (0.956, 0.992)*	0.006*	0.970 (0.951, 0.990)†	0.003†
**Age**				
<50 years	0.998 (0.955, 1.062)	0.803	1.007 (0.953, 1.050)	0.865
50-60 years	0.953 (0.925, 0.982)	0.002	0.952 (0.925, 0.979)	<0.001
≥60 years	0.980 (0.953, 1.009)	0.149	0.977 (0.952, 1.000)	0.074
**Sex**				
Female	0.967 (0.937, 0.998)	0.036	0.964 (0.933, 0.997)	0.035
Male	0.978 (0.955, 1.000)	0.059	0.972 (0.949, 0.996)	0.023
**BMI (Kg/m^2)**				
Non-obese	0.976 (0.956, 0.996)	0.019	0.975 (0.955, 0.997)	0.024
Obese	0.959 (0.910, 1.011)	0.115	0.969 (0.917, 1.020)	0.251
**Smoking status**				
Never	0.984 (0.954, 1.011)	0.304	0.969 (0.941, 0.998)	0.039
Ever smoker	0.970 (0.947, 0.993)	0.011	0.971 (0.950, 0.993)	0.010

* Model 1: adjusted for age and sex.

† Model 2: adjusted for age, sex, ethnicity (white/other), education (None, GCSE/O-level/CSE,A-levels/HNC/HND/NVQ, University degree, Other), deprivation index, fruit and vegetable intake, smoking status (never, former smoker, current smoker) and alcohol intake status (never, former drinker, current drinker).

Supplementary Table S8. Associations between physical activity (per SD increase) and site-specific cancer risk in primary and 2-year lag Cox proportional hazards models

Cancer Type	Cases	HR per sd (95% CI)*	*P**	P_{FDR}*	HR per sd (95% CI)†	*P*†	P_{FDR}†
Head and neck	96	0.913 (0.651, 1.280)	0.596	0.905	0.927 (0.661, 1.301)	0.662	0.946
Oesophagus	102	0.714 (0.519, 0.983)	0.039	0.104	0.771 (0.563, 1.055)	0.104	0.243
Colorectum	561	0.854 (0.767, 0.95)	0.004	0.032	0.86 (0.772, 0.958)	0.006	0.046
Pancreas	133	0.925 (0.714, 1.198)	0.554	0.719	0.937 (0.723, 1.214)	0.621	0.758
Lung	287	0.759 (0.612, 0.942)	0.012	0.047	0.860 (0.700, 1.057)	0.151	0.29
Melanoma	98	1.005 (0.890, 1.135) [a]	0.931	0.958	1.017 (0.839, 1.233) [a]	0.859	0.945
Breast	957	0.872 (0.800, 0.950) [a]	0.002	0.031	0.868 (0.795, 0.947) [a]	0.002	0.036
Endometrium	150	0.802 (0.625, 1.030) [b]	0.084	0.156	0.787 (0.61, 1.016) [b]	0.066	0.194
Ovary	101	0.580 (0.403, 0.836) [b]	0.003	0.032	0.593 (0.411, 0.856) [b]	0.005	0.046
Prostate	1,293	1.050 (0.996, 1.108)	0.073	0.151	0.937 (0.723, 1.214)	0.621	0.758
Kidney	116	0.676 (0.524, 0.872)	0.003	0.032	0.703 (0.546, 0.906)	0.006	0.046
Bladder	99	0.783 (0.652, 0.941)	0.009	0.041	0.821 (0.685, 0.984)	0.032	0.135
Non-Hodgkin lymphoma	213	0.774 (0.641, 0.935)	0.008	0.041	0.759 (0.627, 0.919)	0.005	0.046
Multiple myeloma	100	0.721 (0.528, 0.985)	0.04	0.104	0.712 (0.518, 0.977)	0.035	0.135
Leukaemia	99	0.771 (0.598, 0.993)	0.044	0.111	0.806 (0.627, 1.037)	0.094	0.243

* Models adjusted for age and sex.

† Models adjusted for age, sex, ethnicity (white/other), education (None, GCSE/O-level/CSE,A-levels/HNC/HND/NVQ, University degree, Other), deprivation index, fruit and vegetable intake, smoking status (never, former smoker, current smoker) and alcohol intake status (never, former drinker, current drinker).

[a] Additional covariates specific to melanoma included frequency of sun/UV protection (never/rarely/sometimes, most of the time/always, do not go out in sunshine).

[b] Additional covariates specific to breast cancer included HRT use (ever/never used), oral contraceptive use (ever/never used), number of live births, age at menarche (<12, 12–14, ≥15 years), age at menopause (<45, 45–55, ≥55 years, not yet/unsure), hysterectomy status.

Supplementary Table S9. Assessment of horizontal pleiotropy and heterogeneity in Mendelian randomization analyses

Cancer type	*Cochran's Q*	P_{het}	Egger intercept	SE	P_{ple}
Total cancer	132.8	<0.001	−0.171	0.093	0.140
Head and neck	4.5	0.480	-0.175	0.251	0.525
Oesophagus	7.48	0.187	0.061	0.176	0.747
Colorectum	11.98	0.035	0.004	0.116	0.974
Pancreas	2.7	0.747	-0.157	0.076	0.109
Lung	3.35	0.646	-0.003	0.068	0.970
Melanoma	8	0.156	-0.07	0.108	0.551
Breast	19.16	0.002	-0.002	0.085	0.986
Endometrium	4.9	0.428	0.015	0.117	0.904
Ovary	9.3	0.098	0.048	0.181	0.805
Prostate	9.37	0.095	0.034	0.073	0.661
Kidney	5.77	0.329	0.223	0.173	0.268
Bladder	5.43	0.366	-0.031	0.093	0.752
Non-Hodgkin lymphoma	5.92	0.314	-0.025	0.149	0.872
Multiple myeloma	2.55	0.768	-0.059	0.114	0.633
Leukaemia	14.86	0.011	-0.211	0.255	0.455

Cochran's Q: Cochran’s Q statistic value estimated by heterogeneity test

P_{het}: P value of heterogeneity test

P_{ple}: P value of pleiotropy test based on MR-Egger intercept

Supplementary Table S10. Sensitivity analyses for the causal effect of MVPA on total cancer risk using alternative Mendelian randomization methods

Methods	Causal Effect	95% CI	*P*
2SRI	0.977	0.962 to 0.992	**0.002**
Bayesian-MCMC[a]	0.997	0.994 to 0.999	-
IVW	0.997	0.996 to 0.998	**<0.001**
Egger	1.011	0.934 to 1.095	0.148
Weighted median	0.925	0.878 to 0.975	**0.004**

2SRI: Two-Stage Residual Inclusion.

[a] The credible interval, instead of P value, was used to make statistical inference for Bayesian analysis.

Supplementary Table S11. Linear mendelian randomization estimates for analysis of MVPA on specific cancer incidence

Cancer Type	Cases	HR (95% CI)	*P*	P_{FDR}
Head and neck	103	0.831 (0.504, 1.369)	0.467	0.570
Oesophagus	93	0.528 (0.312, 0.895)	**0.018**	0.054
Colorectum	427	0.926 (0.868, 0.987)	**0.018**	0.054
Pancreas	128	0.860 (0.560, 1.323)	0.494	0.570
Lung	301	0.736 (0.647, 0.839)	**<0.001**	**<0.001**
Melanoma	127	1.056 (0.985, 1.131)	0.126	0.236
Breast	842	0.960 (0.926, 0.995)	**0.024**	0.061
Endometrium	102	0.912 (0.563, 1.478)	0.708	0.708
Ovary	84	0.735 (0.436, 1.240)	0.249	0.340
Prostate	1,197	0.773 (0.301, 1.985)	0.593	0.637
Kidney	103	0.714 (0.634, 0.805)	**<0.001**	**<0.001**
Bladder	106	0.704 (0.640, 0.775)	**<0.001**	**<0.001**
Non-Hodgkin lymphoma	213	1.262 (0.883, 1.803)	0.201	0.302
Multiple myeloma	86	0.868 (0.507, 1.486)	0.606	0.649
Leukaemia	134	0.732 (0.479, 1.120)	0.151	0.251

Supplementary Table S12. Nonlinear Mendelian randomization estimates for MVPA and site-specific cancer incidence

**Cancer Type**	**Cases**	**Threshold**	***P***	$P_{nonlinear}$
Oesophagus	93	293.5	0.071	0.130
Colorectum	427	293.0	0.078	0.124
Lung	301	293.4	0.039	0.208
Breast	842	290.8	0.046	0.487
Prostate	1,197	294.6	0.521	0.532
Kidney	103	293.4	0.378	0.406
Bladder	106	293.4	0.017	0.398
